# The breastfeeding experience of women with major difficulties who use the services of a breastfeeding clinic: a descriptive study

**DOI:** 10.1186/1746-4358-3-17

**Published:** 2008-08-05

**Authors:** Caroline Lamontagne, Anne-Marie Hamelin, Monik St-Pierre

**Affiliations:** 1 Public Health Office in the Capitale-Nationale, Québec, G1E 7G9, Canada; 2 Department of Food Sciences and Nutrition, Université Laval, Québec, G1V 0A6, Canada

## Abstract

**Background:**

Many women experience breastfeeding difficulties. Sometimes these difficulties lead to breastfeeding cessation. Breastfeeding clinics provide support for women facing such problems. This study aims to describe the breastfeeding experience of women, particularly those who use the services of the breastfeeding clinic located in the Greater Quebec City area.

**Methods:**

This is a descriptive study based on information gathered through telephone questionnaires that were administered in 2006 to a sample of 86 women and semi-structured interviews conducted with 12 of these women.

**Results:**

Painful nipples/breasts, low milk supply and latching difficulties were the three most frequent major breastfeeding problems identified by women. Their personal characteristics as well as the moral and physical support provided by family and friends and by health professional and clinicians at the breastfeeding clinic were the factors identified most often as having a positive influence on the breastfeeding experience.

**Conclusion:**

The results suggest that breastfeeding clinics have a critical role to play in improving the breastfeeding experience of women with major difficulties.

## Background

The Ministry of Health and Social Services in the province of Quebec, Canada, has established the following breastfeeding targets for 2007: 85% of infants breastfed at hospital discharge; 70%, 60% and 50% of infants breastfed at two, four and six months respectively; and 20% of infants breastfed at one year [1]. A recent report indicated that the prevalence of any breastfeeding in Quebec in 2005 was close to these goals, with a rate of 85% at hospital discharge and 47% at six months [2]. Breastfeeding rates for the Greater Quebec City area were similar to those for the province as a whole [2]. However, these rates were still far from those of other industrialized countries such as Norway (80% at six months) and Sweden (70% at six months) [3]. In addition, exclusive breastfeeding rates for the province of Quebec (3% at six months) [2] remain far below the 10% goal set by the Ministry [1].

Health is a provincial jurisdiction in Canada, which means Quebec is in charge of determining which health services it offers, including those related to breastfeeding. Since Quebec has a public healthcare system and public health insurance, most health services are provided free of direct charge or at minimal cost. Breastfeeding services normally available in Quebec include prenatal courses and systematic one-time postnatal home visits by nurses from local community services centers. In some regions, including Greater Quebec City and Trois-Rivières, nurses may make more than one visit if necessary, for example in the event of breastfeeding difficulties. Breastfeeding support groups are also active in most regions of Quebec. Lastly, several newly created breastfeeding clinics staffed by lactation consultants and general practitioners have been established in the province (Lapointe and Martel, regional breastfeeding health officers, personal communication, 2008).

A study of 407 women in the Greater Quebec City area in the late 1990s revealed that many women experienced problems including cracked nipples, low milk supply, latching difficulties or breast refusal, blocked ducts, hungry baby, frequent feedings, frequent baby crying, mastitis, and sore nipples [4]. Among the main reasons given by these women for cessation of breastfeeding were problems such as milk insufficiency (perceived or real), weight issues of baby (insufficient weight gain), lack of time for the mother, and breast problems [4].

Different programs and interventions aim to promote breastfeeding or overcome breastfeeding difficulties. Scientific evidence indicates that among the interventions shown to be beneficial for prolonging breastfeeding duration, breastfeeding support by skilled peers and professionals is effective when provided to women in a proactive way. A combination of information, support and guidance, as well as unrestricted mother-baby contact and unrestricted feeding are other effective interventions [5-9].

Few studies of breastfeeding clinics in industrialized countries have been identified [10-17]. These clinics aim to increase breastfeeding duration and diminish the prevalence of difficulties [10,11,14]. According to the research findings, women were satisfied with these clinics. They felt that the clinics provided valuable knowledge about breastfeeding and could help them prolong the duration of breastfeeding and find solutions to the difficulties they experienced [10,11,14,15].

At the time of this study, there was one breastfeeding clinic in Quebec City, located at the *Centre Hospitalier Universitaire de Québec *(CHUQ). It was established in 2004 by the Quebec City regional public health department and the CHUQ at the request of field workers who wanted to provide better support to women with acute breastfeeding problems. At that time, the clinic was funded in part by the regional public health department and in part by the CHUQ [18]. Referrals are mandatory to access the services of the clinic. Women using the facility are seen by an International Board Certified Lactation Consultant (IBCLC) and a physician. According to the satisfaction forms collected in 2004–2005, women were highly satisfied with the clinic for various reasons, including a warm reception, waiting time, usefulness of the information and treatments received, and overall satisfaction with their experience at the clinic [18].

This paper aims to describe the breastfeeding experience of women experiencing major breastfeeding difficulties and in particular, the experience of women using the services of the Quebec City Breastfeeding Clinic.

## Methods

### Design

This descriptive study conducted in 2006 used a mixed methodology. The quantitative component comprised a telephone questionnaire and statistical analysis of the results while the qualitative component consisted of individual semi-structured interviews and a content analysis of the verbatim interview transcripts. This study was part of a larger research project which aimed to evaluate the effects of the Quebec City Breastfeeding Clinic on breastfeeding duration and satisfaction among women in comparison with women who did not receive clinic services [19].

### Sample

This study was conducted with French speaking women aged 20 years or older having experienced major breastfeeding difficulties. These women were from two regions – the Greater Quebec City area and Trois-Rivières – where breastfeeding rates are similar [2]. The women living in the Greater Quebec City area had used the services of the Quebec City Breastfeeding Clinic. The women from Trois-Rivières, a town located 200 km southwest of Quebec City, had had access to standard breastfeeding services, as described in the background section above, but not to a breastfeeding clinic.

To be included in the study, women had to be primiparous, have given birth to a healthy, full-term baby, and have experienced major problems with breastfeeding during the first two months of their baby's life. Problems were considered major if they were serious enough to compromise ongoing breastfeeding and if all steps had been taken in the course of normal follow-up to try to solve them. Examples included excessive pain during latching (possible causes: thrush, cracked nipples, vasospasms, breast infections); infant weight loss that could potentially compromise baby's health (possible causes: low milk supply, improper latching, breast refusal, difficulty staying latched); and baby's inability to breastfeed (possible causes: all of the above, as well as other problem situations such as tongue tie (ankyloglosia)). Women from Quebec City had to have given birth between July 1, 2004, and August 31, 2005. Given the smaller population of Trois-Rivières, the period was extended from May 1, 2004, to December 31, 2005, for women from this city. Women who had given birth to an infant born with an abnormality or major illness were not included in the study.

Women from Quebec City were recruited by means of a systematic sampling method with a random start: an initial record was randomly chosen from the list of all the records of children having attended the breastfeeding clinic in the given period, followed by every fourth record in sequence, stratified for the locations (2) where the clinic was held. Once eligibility criteria were checked, a member of the research team (MSP) phoned the mothers to invite them to participate in the study. For Trois-Rivières, the women were identified from all birth records for the eligibility period kept at local community service centers. All those who satisfied the selection criteria were contacted by phone by a member of the research team (MSP). It was also determined at this time whether they had experienced major breastfeeding difficulties. All the women from Trois-Rivières who met the eligibility criteria and agreed to participate were included in the study.

The subsample of women who participated in semi-structured interviews in both cities was selected using a purposeful sampling method designed to maximize variations in breastfeeding duration (0 to 12 months and more) and the education level of participants (vocational diploma or less to university degree or more).

The sample for the study, which was conducted as part of a Master's Degree program, is relatively small: approximately 50 women per group for the telephone survey and 6 women per group for the interviews.

### Instruments and data collection

#### Telephone questionnaire

The questionnaire was pre-tested with five women from the Greater Quebec City area. After readjustments, it was administered to women from both cities. The questionnaire comprised 80 questions and examined sociodemographic, economic, clinical, and psychosocial characteristics, as well as the support respondents received, their breastfeeding experience, and their experience at the Quebec City Breastfeeding Clinic. The majority of questions were drawn up specifically for the study. However, the wording for certain questions like those on sociodemographic characteristics of the mother were based on those used by major Canadian and Quebec studies [20-25]. Other questions, notably those on breastfeeding, were based on a telephone questionnaire used in an earlier study conducted in the Greater Quebec City area in 1998 [4]. Questions on the respondents' level of satisfaction with their breastfeeding experience, the healthcare professionals involved, and the interventions they received were measured on a scale of one to five (1 = highly dissatisfied, 5 = highly satisfied). See additional file 1: *telephone questionnaire *for themes from the questionnaire and for sample questions. A survey firm administered the telephone questionnaire to all the women. It was decided to use a survey firm to minimize the length of the data collection period. The firm chosen (SOM Surveys, Opinion Polls and Marketing) has a good reputation in the Quebec health research field. Interviewers received training on the study context and the questionnaire in the presence of a member of the research team (CL). Administering the telephone questionnaires took around 20 minutes per respondent.

#### Semi-structured interview

In this study, data from the qualitative component provided information that allowed for a better understanding of the breastfeeding experience [26]. The interview scenario included 17 questions on the respondent's personal history of breastfeeding, facilitating factors and obstacles to breastfeeding, social support, the experience at the Breastfeeding Clinic, the respondent's opinion about a potential clinic for Trois-Rivières and other relevant information related to the breastfeeding experience (see additional file 2: Interview grid). The individual interviews where conducted by a member of the research team (CL) at Université Laval for the women in Quebec City, and at a local health and community services center for the women of Trois-Rivières. The one hour interviews were taped and transcribed.

### Ethical considerations

Before administering the telephone questionnaire, interviewers explained the study to the women. A consent form was read aloud and women who wanted to participate gave their verbal consent. The same process applied to the semi-structured interviews, but with written consent. The study was approved by the Clinical Research Ethics Committee at Centre Hospitalier de l'Université Laval. Ethical approval was also obtained from the Health and Social Services Center in Trois-Rivières.

### Data analysis

The responses to the telephone questionnaire were described using frequency tables. Chi-square and Fisher's exact tests were employed to compare proportions of general characteristics of participants (age, marital status, education, and income) by their city of origin. A probability level of < 0.05 was used to establish statistical significance. SPSS 13.0 software for Windows 2004 [27] was used to perform the quantitative analysis.

The semi-structured interviews were fully transcribed, then subjected to a content analysis [28]. This aimed to determine the meaning of the message studied, and consisted of classifying the transcribed elements and codifying them in various categories in order to bring out the different characteristics and achieve better understanding of the exact meaning. L'Écuyer general procedures for content analysis were applied [28]: 1) preliminary reading and establishment of a list of statements; 2) choice and selection of units of classification; 3) categorization and classification; 4) quantification and statistical processing of the data; 5) scientific description; and 6) interpretation of the results. In the framework of this study, step four was omitted. Step three involved mixed categories, with pre-determined categories based on scientific literature and others based on emerging data. NVivo 2.0 software, 2002 [29] was used to help code the data. An intracoder reliability measure was performed using the Miles and Huberman formula [30]. This showed a percentage of 74% and after discussion, a percentage of 100%.

**Figure 1:**
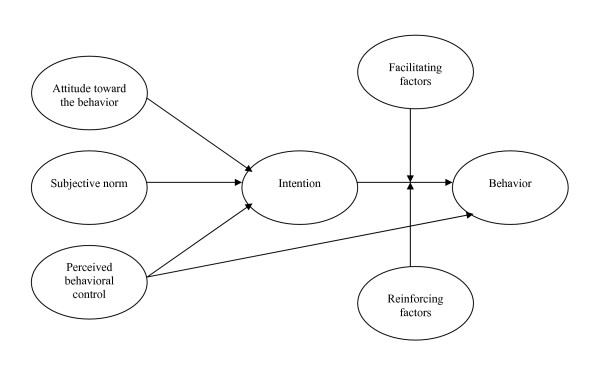
Conceptual framework.

Conceptual benchmarks were used to support the analysis. The theory of planned-based behavior had already been found useful for studying breastfeeding determinants [31-33]. According to this theory [34], behavior is determined by intention and perceived behavioral control. Intention is self-determined by attitude, subjective norms, and perceived behavioral control. Two other concepts of this model, facilitating factors and reinforcing factors, were added by Godin [35-37]. For this study, Godin's conceptual framework (Figure 1) served to develop interview questions that would help identify factors influencing women's experience of breastfeeding. It subsequently served to organize the interview data and also facilitated understanding of the influencing factors in the discussion of interview results.

## Results

### Description of the sample

One hundred and forty women were selected to take part in this study. Of the 72 women selected from the Greater Quebec City area, 13 could not be reached, five refused to participate for reasons unknown, and two were excluded because they did not meet eligibility criteria. This left a subtotal of 52 women, including the five women who took part in the pretest; their responses were counted, except for answers to five questions that had been considerably modified after the pretest. For Trois-Rivières, 68 women were selected for the study. Of this number, 27 could not be reached, including 20 whose eligibility for the study was unknown. Three women refused to participate and four others were excluded, leaving a total of 34 participating women. Of the 86 women who completed the study, 12 took part in the semi-structured interviews, six from Greater Quebec City and six from Trois-Rivières. The final refusal and loss rate was 28% for Greater Quebec City and 50% for Trois-Rivières.

The sociodemographic and economic characteristics of the participants, namely age, marital status, education level, and family income, shown in Table 1 suggest no baseline differences between the two groups.

**Table 1:** Sociodemographic characteristics

**Characteristics**	**Total (n = 86) **n (%)	**Québec (n = 52) **n (%)	**Trois-Rivières (n = 34) **n (%)	**p value**
**Maternal age**				0.94**
20 to 29 years	48 (56)	29 (56)	19 (56)	
30 to 34 years	24 (28)	14 (27)	10 (29)	
35 years and more	14 (16)	9 (17)	5 15)	

**Marital status**				0.15*
Married/common-law	84 (98)	52 (100)	32 (94)	
Divorced	1 (1)	0 (0)	1 (3)	
Single/never married	1 (1)	0 (0)	1 (3)	

**Education**				0.40*
Vocational diploma or less	11 (13)	7 (13)	4 (12)	
College diploma	28 (33)	14 (27)	14 (41)	
University diploma	47 (55)	31 (60)	16 (47)	

**Family income**				0.39*
< 30 000$	9 (10)	4 (8)	5 (15)	
30 000 to < 50 000$	17 (20)	9 (17)	8 (24)	
≥50000$	60 (70)	39 (75)	21 (62)	

* Fisher's exact test

** Chi-square test

### Description of the breastfeeding experience

According to the data collected with the telephone questionnaire, women reported experiencing several types of breastfeeding difficulties as presented in Table 2. Painful nipples/breasts, low milk supply, insufficient infant weight gain, absence of infant bowel motions, and latching problems and breast refusal were the most frequent breastfeeding problems identified by women.

**Table 2:** Breastfeeding difficulties experienced

Difficulties**	Total (n = 79)* n (%)
Painful breast/nipple	70 (89)
Milk insufficiency/insufficient weight gain/stool absence	51 (65)
Latching/breast refusal	37 (47)
Sucking difficulties	12 (15)
Frequent baby crying	8 (10)
Inverted nipple	5 (6)
Other	3 (4)

* Data are missing for 5 women from the pretest (wording of the pretest question was different and we have decided not to keep their answers) and for 2 women who did not answer this question but indicated that they had breastfeeding difficulties in another question.

** Each woman could provide from 1 to 3 difficulties.

Reasons for cessation of breastfeeding were also similar for women: latching problems or breast refusal, low milk supply, and pain were the most frequent reasons (Table 3). Other important reasons for cessation were the return to work, school, or daycare and the attainment of breastfeeding objectives or the belief that the infant was old enough to stop breastfeeding.

**Table 3:** Reasons for ceasing breastfeeding

Reasons**	Total (n = 75)* n (%)
Latching difficulties/breast refusal	29 (39)
Low milk supply	28 (37)
Pain	20 (27)
Return to work/school or start of daycare	15 (20)
Breastfeeding objective attained/child old enough	14 (19)
Inconvenience/tired from breastfeeding	10 (13)
Child's health/weight loss	10 (13)
Lack of time/need for autonomy/freedom	5 (7)
Health problem in mother/medication	4 (5)
Desire to drink alcohol/varied diet	2 (3)
Child's desire or choice	2 (3)
Person's opinion	1 (1)
Effect of bottle	3 (4)
Other linked to woman's morale	3 (4)
Other	10 (13)

* Data are missing for 5 women from the pretest (wording of the pretest question was different and we have decided not to keep their answers) and for 4 women who had not ceased breastfeeding at the time the questionnaire was conducted.

** Woman could provide from 1 to 3 reasons.

Information gathered during the 12 individual interviews (see Table 4 for a list of the main categories) also showed that some women experienced other, more specific problems. In certain cases, these affected them personally. Examples included fatigue, anemia, and significant postpartum pain: "I'd had a big episiotomy, I had anemia [. . .] and I was really weak. I don't think that was much help either" (QC6-82 – 10 days. Quote is our translation as are all subsequent quotes in this paper, and the period refers to the baby's age at the time of breastfeeding cessation). In other cases, infant health problems were the issue: " [. . .] he was too weak. His little heart was weak, his breathing weak, I didn't have a choice. I was feeding five minutes per breast, then I had to stop and quickly give him bottled milk" (QC5-21 – 3 months). Many of the women who experienced these difficulties also affirmed having felt sadness, discouraged and concern at the time: "The breastfeeding wasn't going all that great, and I felt really sad because I didn't want to give up." (QC1-16 – 15 months) Potential links between the difficulties they experienced and their physical well-being were not addressed by the women.

**Table 4:** List of principal content analysis categories, illustrated with verbatim excepts

**Breastfeeding history**
**Breastfeeding difficulties**
"The third week at home, she was having trouble latching on to the breast." (QC1-16)*
**Breastfeeding duration**
*"I breastfed full time for ten days."(TR3-8)*
**Satisfaction with the breastfeeding experience**
*"As far as the length of time is concerned, I'm very satisfied" (QC2-12)*
**Cessation of breastfeeding**
*"The only reason I stopped was because of the pain." (TR6-77)*
**Influencing factors**
**Human**
**Formal **(e.g. nurses, physician, others)
*"The nurses are there and they help you." (QC1-81)*
**Informal **(e.g. partner, mother, friends, others)
*"My mother helped me a lot." (QC5-97)*
**Factors relative to the mother **(e.g. intention, personality, others)
*"I was really determined to breastfeed my baby." (TR2-18)*
**Social pressure**
*"Because I really wanted to breastfeed, because everyone was talking about it and pushing me." (TR1-12)*
**Others **(e.g. maternity/paternity leave, locations and facilities, others)
*"There are nursing rooms everywhere." (QC1-42)*

**Breastfeeding clinic**
**Services and interventions received**
*"That's it, they watched how I put E to the breast." (CQ1-162)*
**Satisfaction with the clinic**
*"The staff was friendly and efficient." (QC5-240)*
**Utility of the breastfeeding clinic**
*"They were such a big help. It seems to me that if anyone else had breastfeeding problems, it would be much easier for her to consult them." (QC2-212)*
**Influence of the clinic on the breastfeeding experience**
*"How did it influence me to continue, yeah, that's it, with those medications. To see if it would stimulate my milk let-down." (QC4-190)*

Quote is our translation as are all subsequent quotes in this table.

In addition, although some women were satisfied with their experience and expressed no regrets for having ceased breastfeeding, many of those interviewed expressed disappointment, sadness, and regret in having stopped: " [. . .] I was sad, I cried a lot. It lasted almost a month. A good four weeks after I'd stopped breastfeeding, I cried. At night, I'd go to bed and shed a little tear" (QC5-86 – 3 months). For others, cessation came as relief, since with bottle feeding, infants would gain weight and women would be less tired: "It was really a big relief [. . .] and it was overnight. Once I made my decision, everything was fine afterwards. I felt better" (TR3-56 – 6 months).

Satisfaction with regards to the breastfeeding experience differed among the women who took part in the interviews. For almost half of them, it was a wonderful experience and they were very satisfied. As one woman said: "I'm totally satisfied, and if I have another child, I'll do it again for sure" (QC5-70 – 3 months). Others, especially those unable to overcome their difficulties, found the experience difficult and felt dissatisfied and disappointed: "But that's the thing, you see, part of me was dissatisfied because [. . .] my problem never really got solved" (TR6-42 – 6 weeks). Half of the women even felt guilty for ceasing breastfeeding or perceived cessation as a failure, as the following accounts show: "But you feel really guilty, too, because you've read everywhere that it's the best thing, so it's really tough. You say to yourself, man, and you feel guilty" (QC6-12 – 10 days). "I took it as a failure, because I wanted to give this child the best of everything. For me, it was a failure not to live up to my ideal" (TR2-43 – 2 months).

### Personal influences

During the interviews, three women affirmed that they themselves were the most important person influencing their own breastfeeding experience: "I'd have to say I was [the most important person]" (TR5-116-118 – 6 months). In fact, a wide variety of personal characteristics or aspects could influence a woman's experience. First of all, intention to breastfeed was a major factor. From the beginning, certain women firmly intended to breastfeed their infant, sometimes even having a set goal in mind for the duration: "I had this idea in mind that I would breastfeed her for six months" (QC2-70 – 8 months). The firmness of these women's intentions gave them the motivation to traverse their difficulties and reach their goals. As one woman put it: "No, I wanted it so much (laugh). Everything that looked like an obstacle, I got rid of it. For me, breastfeeding came first and everything else was secondary, so, I don't know what else could have stopped me from breastfeeding" (QC5-162 – 3 months). Other women, however, were less certain regarding their intention to breastfeed and were clearly not as motivated: "Anyway, when I was pregnant, I wasn't sure I wanted to breastfeed" (TR1-40 – 3 days).

Women's attitudes toward breastfeeding were also important. Almost all the women mentioned the benefits of breastfeeding for society, infants, and women. They also mentioned the practical aspects of breastfeeding and its low cost, as well as other factors influencing the intention to initiate or continue breastfeeding as expressed in this statement: "It's always ready, it's always at the right temperature, there's nothing to sterilize. No matter where you are, you can always breastfeed" (QC1-12 – 15 months).

In addition, in light of what women said during the interviews, determination and perseverance appear to have influenced their experience: " [. . .] I told myself, 'Hey, I can do this, too. I want to breastfeed, I've got enough milk, the baby's feeding well, there's no reason to stop"' (TR6-65 – 6 weeks). Determination helped certain women to continue breastfeeding, even though some of them had told themselves at the beginning that they would not persist if they found it was not working: "It wasn't really working for me, but I really stuck with it" (TR2-39 – 2 months). Their determination and perseverance helped them overcome the effects of health problems and tiredness. One woman said, "Because of how tired I was, I wouldn't have been able to handle breastfeeding any longer. I was exhausted" (QC6-71 – 10 days). Hope that problems would cease and that breastfeeding would become easier over time helped other women to continue breastfeeding: "At the start, what really kept me going was the constant hope that things would get better, and that it wouldn't hurt as much" (TR6-34 – 6 weeks). Some women expressed discomfort and shyness that sometimes led them to refrain from breastfeeding in public: "I'm not necessarily all that comfortable about breastfeeding in public" (TR3-100 – 2 weeks).

Most women really appreciated the contact, closeness, and bonding with their infant during breastfeeding. For some women, this contact was a motivation to pursue breastfeeding as this statement shows: "I liked the contact with the baby [. . .]. So that's why I kept at so long, because if I had listened to myself, just from a physical standpoint, I would have breastfed for one week, than stopped" (TR6-34 – 6 weeks). However, for one woman, the contact was spoiled by some major difficulties and ceased to be a motivation factor: "So I'd reposition the baby, bring it back to the breast, try again, and all that time I'd forget about contact [. . .]. That lovely moment became pretty technical, and that took away some of the charm" (QC6-36 – 10 days).

### Social influences

Apart from aspects directly related to the women themselves, other factors such as social support contributed to their breastfeeding experience. According to the telephone questionnaires, as shown in Table 5, women's partners were the ones who encouraged and supported them the most throughout their experience. They were followed by nurses from local community services centers who visited the women in their homes, by clinicians at the breastfeeding clinic (named only by women from Quebec City) and then by mothers, friends, and hospital nurses.

**Table 5:** Persons who had most supported and encouraged women throughout breastfeeding experience

Persons**	**Total ** (n = 81)* n (%)
Partner	54 (67)
Nurse from local community services centers	32 (40)
Personnel of breastfeeding clinic	25 (31)
Mother	21 (26)
Hospital nurse	20 (25)
Friend	19 (23)
Volunteer from a breastfeeding group	13 (16)
Physician (other than breastfeeding clinic one)	11 (14)
Family member (not partner or mother)	10 (12)
Midwife	1 (2)
Other	6 (7)

* Data are missing for women (5) from the pretest (wording of the pretest question was different and we have decided not to keep their answers).

** Woman could provide from 1 to 3 people.

According to the semi-structured interviews, social support – whether formal (health professionals) or informal (partner, family and friends) – included both moral and physical support. Moral support was expressed through encouragement, reassurance, listening and a positive attitude, all of which helped reinforce the women's determination to pursue breastfeeding. For example, one woman said: "It was because she was a nurse who really believed in breastfeeding. She'd say, 'Don't give up.' She would encourage us all the time and she'd push us to continue" (TR4-32 – 3 weeks). However, men could undermine the women's determination if they did not support their partner in the pursuit of breastfeeding as suggested by this following account: "My husband was not all that encouraging, because like I said before, he wanted to participate and was looking forward to giving the bottle" (TR1-59 – 3 days). Health professionals could also have a negative influence when their comments and attitudes were discouraging or made women feel guilty, or when their attitudes were too pro-breastfeeding or lacked tact. One woman made the following remark which also hints at the fragility of women at this particular time of their life: "Sometimes they (the nurses) can be a bit insensitive, I found they weren't always tactful" (TR1-67 – 3 days).

Physical support included advice and tips related to breastfeeding, help with breastfeeding management, and help with housework (cleanup, cooking, etc.), as described in statements like these: "They (the hospital nurses) helped at the beginning, you know, with positions for feeding, right away at the hospital" (TR3-66 – 2 weeks). "My mom came to the house to give me a hand. It could be a bit overwhelming at times, so she'd clean up, do the wash, do a bit of cooking. It was a big help" (QC2-97 – 8 months). Nevertheless, in cases where health professionals did not detect a problem or gave conflicting or erroneous advice, they hampered the breastfeeding experience, as the following comment suggests: "One (nurse at the hospital) has her technique, another has hers. So you do what the last one told you, and then you're told it's wrong" (TR1-67 – 3 days).

Furthermore, two women felt that they had been left to themselves after returning home, or did not use the services available. This may have had a negative effect on their experience. One of them said: "No one came to see me to give me tips, help out, or show me positions. I was kind of left to myself, but I didn't ask for help either" (TR1-59 – 3 days).

In addition to the influence of support on the breastfeeding experience, the interviews also revealed that women felt significant social pressure to breastfeed. Some women explained that it was mostly the positive aspects of breastfeeding that were promoted, and that the more difficult aspects were not always addressed. For almost half the women, there was so much information related to breastfeeding that they felt obligated to breastfeed and hesitated to stop because of all the pressure. One women said: "I felt that I was not a good mother if I was not breastfeeding [. . .] I found there was a lot of pressure. Social pressure" (TR1-28 – 3 days). However, this pressure was never mentioned in relation to the breastfeeding clinic.

### Breastfeeding clinic influences

Breast or nipple pain, latching difficulties or breast refusal, low milk supply, and insufficient weight gain and sucking difficulties were the main reasons evoked by the study participants for consulting the Quebec City Breastfeeding Clinic, as shown in Table 6.

**Table 6:** Reasons for consultation at the breastfeeding clinic

Reasons for consultation**	Greater Quebec City (n = 47)* n (%)
Painful breast/nipple	30 (64)
Latching problems/breast refusal	24 (52)
Milk insufficiency/insufficient weight gain	14 (30)
Sucking difficulties	10 (21)
Frequent baby crying	4 (9)
Inverted nipples	2 (4)

* Data are missing for 5 women from the pretest (wording of the pretest question was different and we have decided not to keep their answers).

** Each woman could provide from 1 to 3 reasons.

Women seemed highly satisfied with the clinic. As presented in Table 7, the telephone questionnaire shows that the majority (over 85%) of women were satisfied or highly satisfied with the services and interventions provided by the clinic and the clinicians, either the IBCLC or the physician. The participants also believed that the clinic had helped them to reach (75%) and even surpass (48%) their breastfeeding objectives (Table 7). In addition, they believed that it had increased their satisfaction with their overall breastfeeding experience (85%).

**Table 7:** Women's satisfaction with the breastfeeding clinic and the effect of the clinic on attainment of their objectives and their satisfaction

	**Greater Quebec City (n = 52) **n (%)
Satisfaction* with the services and interventions of the clinic	
Very dissatisfied/dissatisfied (1–2)	2 (4)
Moderately satisfied (3)	4 (8)
Satisfied/very satisfied (4–5)	46 (88)

**Satisfaction with the physicians of the clinic**	
Very dissatisfied/dissatisfied (1–2)	2 (4)
Moderately satisfied (3)	4 (8)
Satisfied/very satisfied (4–5)	43 (88)
Missing data: 1	

**Satisfaction with the lactation consultants of the clinic**	
Very dissatisfied/dissatisfied (1–2)	1 (2)
Moderately satisfied (3)	2 (4)
Satisfied/very satisfied (4–5)	44 (94)
Missing data: 5	

**Clinic allowed them to reach their breastfeeding objectives**	
Yes	39 (75)
No	12 (23)
Doesn't know	1 (2)

**Clinic allowed them to surpass their breastfeeding objectives**	
Yes	25 (48)
No	14 (27)
Doesn't apply to	12 (23)

**Clinic allowed them to increase overall satisfaction with their breastfeeding experience**	
Yes	44 (85)
No	8 (15)

* Satisfaction was measured on a scale of one to five (1 = highly dissatisfied, 5 = highly satisfied).

During the semi-structured interviews, participants noted that there were two kinds of services offered at the breastfeeding clinic. The first was the identification of the problems that led women to visit the clinic as reported by this woman: "When you arrive, they ask you why you've come. You explain your problem, then they ask you to show them how your baby feeds" (QC4-162 – 3 months). The second was the interventions themselves and the breastfeeding tips given. These included practical advice as well as encouragements, reassurance and explanations related to breastfeeding problems and their possible solutions. For example, women would recount: "He gave me a suggestion to help the baby. It was a little bottle with a tube that I would slip in at the edge of her mouth while she was feeding, because she was drinking less and less milk [. . .] So with that, she had more milk, and her appetite came back" (QC2-10 – 8 months); "It still wasn't working, but at the clinic, they told me 'She's starting, it's not so bad to take the breast away every once in a while at the start of a feeding or in the middle. Don't be discouraged"' (QC3-44 – 4 months).

According to these women, the visits to the clinic had a positive influence on the breastfeeding experience in two ways. The first was in terms of physical support, which included the identification of problems and their resolution or temporary solution. As reported by one participant: "If this advice had not worked, she wouldn't have gained back her weight and I would have put her on the bottle for sure" (QC2-186 – 8 months). The second was in terms of moral support, which included encouragements, reassurance and the positive way in which the clinicians presented the breastfeeding experience. As one woman said: "At the breastfeeding clinic, it was like there was no problem [. . .] They were really reassuring on this. It was 'try, try, everyday [. . .] and you will succeed.' They were really confident" (QC3-79 – 4 months).

In the semi-structured interviews, the participants again expressed their satisfaction with the clinic: "I couldn't have asked for anything more. I found it very suitable, it was perfect for me" (QC5-252 – 3 months). However, women did show some hesitation regarding various organizational aspects, including parking, waiting time, and the fact that the clinic is located in a hospital setting: "There were waits sometimes. You think about the parking when that happens" (QC2-203 – 8 months). However, waiting also had its advantages: "You're never rushed. So the disadvantage of not having your appointment 'on time,' so to speak, works out to your advantage when it's your turn, because you're not hurried and they take time to really listen" (QC3-160 – 4 months). Participants also talked about their satisfaction with the clinicians, who were greatly appreciated for their kindness, empathy, listening skills, warmth, and dedication, as well as for all the information they gave.

The women who attended the clinic unanimously agreed that it was a useful service. They also felt it would be useful for other women experiencing breastfeeding difficulties. As one woman put it: "They were such a big help. It seems to me that if anyone else had breastfeeding problems, it would be much easier for her to consult them. She'd get lots of information and help, plus good support" (QC2-212 – 8 months).

Interestingly, women from Trois-Rivières thought a breastfeeding clinic would be useful for their region because of the staff's specialized expertise and because of the wide range of breastfeeding problems they were familiar with. They felt that there were gaps in the breastfeeding services in their region that required a specialized service like a breastfeeding clinic. One woman said: "For me, I think it would be a good idea to have centers like this in various areas. If we want to continue to promote breastfeeding, it takes support. I mean, there are resources and things here, I'm not trying to say there's nothing, but something a bit more specialized wouldn't hurt" (TR6-129 – 6 weeks).

## Discussion

The women who participated in this study are comparable in terms of age and marital status to a larger group of over 4,000 Quebec women who gave birth in 2005 [2]. However, they appear to be more educated than Quebec mothers on average, with 55% holding university degrees compared to 35% at the provincial level. Family income was also higher, with 70% having a family income exceeding $50,000 compared to 47% for Quebec households in general [2].

The major breastfeeding difficulties experienced by women, such as breast and nipple pain, low milk supply, latching problems, and so on, were similar to those mentioned in a study conducted in the Greater Quebec City area in the late 1990s [4]. In addition, in our study, the reasons for ceasing breastfeeding coincided with the difficulties experienced by other women. These reasons, along with the others mentioned (return to work or school, infant health problems), are comparable to the ones found in other studies in Canada [38], the province of Quebec [39,40], and the Greater Quebec City area [4].

Intentions and attitudes regarding breastfeeding appear to be significant factors influencing the experience. In fact, firm intentions to breastfeed gave women the motivation they needed to face difficulties. According to different studies, prenatal breastfeeding intention is an important determinant of both breastfeeding initiation and duration [33,41-44]. The moment the decision is made, intentions regarding duration seem to be linked with breastfeeding duration [41-43]. Positive attitudes toward breastfeeding have also been associated with the initiation and longer duration of breastfeeding [33,41]. Moreover, these elements correspond to certain concepts from our conceptual framework (Figure 1), which holds that attitudes toward a behavior are predictive of intentions to adopt the behavior, and that those intentions are in turn predictive of actual adoption, i.e., breastfeeding continuation. In addition, determination and perseverance to overcome breastfeeding challenges and difficulties are other notions that have been addressed in other studies [41].

With the exception of the clinicians at the breastfeeding clinic, the sources of social support mentioned by women in this study were similar to those mentioned by women in other studies included in a meta-analysis of qualitative studies [45] and by women in the Greater Quebec City area [4]. These sources include partners, nurses from local community services centers, mothers, friends and hospital nurses. Moreover, a study conducted in the province of Quebec found that women's entourages influenced their decision to breastfeed, and that women generally perceived their entourage as being in favor of breastfeeding [39]. In addition, informal sources of support (partner, family, friends, peers) and formal sources (lactation consultants, nurses, physicians) have been shown to affect both initiation and continuation of breastfeeding [39,41,46].

Unfortunately, as mentioned by some participants in this study, health care professionals can sometimes be a negative influence when they provide women with inconsistent, inaccurate, inadequate, or conflicting breastfeeding information and recommendations [41,45,47-49]. It is therefore important to ensure that health professionals are properly trained with respect to breastfeeding and that women have access to optimal services consistent with the *Baby Friendly Hospital Initiative *and with *The Baby Friendly Initiative in the Community *recommendations [50,51].

Sources of social support and the way they influence the breastfeeding experience are analogous with some of the items of the conceptual framework (Figure 1). In fact, positive support from family and health professionals is similar to the perceived subjective norm influencing intentions to initiate and continue breastfeeding. Advice and interventions provided by a woman's entourage and by health professionals, as well as help with housework, are other facilitating factors, while encouragement and reassurance are reinforcing factors.

The influence of social pressure on the breastfeeding experience was alluded to by three women. In other studies, women have reported feeling pressure both to start and continue breastfeeding against their own wishes [47]. In a qualitative study conducted in England, women also described feeling unprepared for the realities of breastfeeding and said they would have liked to have had more information about the possible inconveniences [52]. Greiner considers that priority should be given to protective and supportive strategies for breastfeeding, rather than to promotion strategies. In fact, he notes that in general, "protective programs put pressure on government and industry, supportive strategies put pressure on the health care system, on networks of women and on employers, while promotion strategies [. . .] can [not easily] avoid putting pressure directly on women themselves" [53]. Because of this, some women in our study may have felt obligated to breastfeed and hesitated to stop. In addition, some women felt guilty for stopping breastfeeding sooner than they planned and perceived this cessation as a failure, as was the case elsewhere [54]. Similarly, an Australian qualitative study found that women may feel confusion, self-doubt and guilt when confronted with incompatible expectations between themselves and other people [48].

Satisfaction rates for the Quebec City Breastfeeding Clinic indicate that over 80% of women in our study were satisfied or highly satisfied with the clinic's staff and services and felt it had helped them increase their satisfaction with their breastfeeding experience. The high satisfaction rates were similar to those recorded for women attending other breastfeeding clinics. At one Canadian breastfeeding clinic in Ontario, over 90% of the 164 respondents reported satisfaction ratings of good or excellent [10]. The clinic helped them feel more confident and positive with regards to their breastfeeding experience, enhance their knowledge of breastfeeding, and prevent or overcome difficulties. Over 70% of the respondents also believed that the clinic had helped them to breastfeed for a longer period [10]. At another Canadian breastfeeding clinic in Saskatchewan, 100% of the 43 respondents were satisfied with the interpersonal aspects of the center and over 90% with the information and support they received. Respondents attributed their satisfaction to the advice given, the participative approach, the quality of information provided, the support, encouragement and reassurance received, and the knowledgeable staff [11]. Similarly, mothers using a breastfeeding clinic in British Columbia, Canada, gave the facility an average rating of 8.7 out of 10 (10 = extremely satisfied) [14]. In an Australian clinic, satisfaction survey showed that most respondents were satisfied with the clinic and felt that the service quality was better than expected [16]. They also responded the staff were professional and knowledgeable in their field of work [16].

A considerable number of women also said that the Quebec City Clinic allowed them to reach or surpass their breastfeeding objectives. They gave the same reasons as those mentioned in other studies [10,11,13-15] to explain the influence of the clinic on the breastfeeding experience, namely, the identification of problems, the solutions found, and the encouragement and reassurance received.

However, it is important to point out that the Quebec City Breastfeeding Clinic does not operate entirely the same way as the majority of the other clinics studied. For example, these clinics employ IBCLC and nurses [10,11,14] or midwives [13,15] while the Quebec City Clinic employs IBCLC and physicians. Also, at the other clinics [10,11,15], women are free to use the services as they wish, while at the Quebec City clinic, women are referred by field workers. Although there are other clinics in Canada that operate in similar ways to the one in Quebec, no scientific papers on their services have been published yet.

One limitation of this study is the small number of participants. Indeed, the number of semi-structured interviews conducted did not allow us to reach content saturation. Another possible limitation is the voluntary interview participation, which may have biased the results in a positive way. The telephone questionnaire and semi-structured interviews used for the study are both retrospective tools that relied on participants' memories. Participants may have forgotten information, which could have biased the results. Furthermore, the two research tools were tested, but not validated.

More research is needed to better understand the breastfeeding experience of women grappling with major difficulties and to better understand why some are able to overcome these difficulties while others are not. It would also be relevant to study how social pressure may positively and/or negatively affect the breastfeeding experience. In addition, more qualitative and quantitative research is needed on women's experience at breastfeeding clinics throughout Canada and in other industrialized countries to better understand the influence of these clinics on breastfeeding issues such as duration and satisfaction.

## Conclusion

This study showed that women's breastfeeding experiences may be influenced by the moral and physical support provided by family and friends, health professionals, and breastfeeding clinic clinicians as well as their own personal traits. These results may be useful for better understanding women's experiences and determining the best ways to help them overcome their difficulties. They may also prove useful in showing the relevance of such clinics for improving the breastfeeding experience.

## Competing interests

The authors declare that they have no competing interests.

## Authors' contributions

This study is part of CL's Masters Degree. She performed all the research steps of this study. AMH participated in the design of the study and supervised all the research steps and writing. MSP participated in the design and coordination of the study. All authors read, commented, and approved the final manuscript.

## Supplementary Material

Additional file 1 Description of the Telephone Questionnaire. Themes from the questionnaire and sample questions. Click here for file

Additional file 2 Interview Grid. Wording of questions of the interview. Click here for file
