# Supplementary material for: The breastfeeding experience of women with major difficulties who use the services of a breastfeeding clinic: a descriptive study
Source: Int Breastfeed J. 2008 Aug 5;3:17. doi: 10.1186/1746-4358-3-17 (PMC2533286; doi:10.1186/1746-4358-3-17)
Supplement: Additional file 1 — Description of the Telephone Questionnaire. Themes from the questionnaire and sample questions. [file 1746-4358-3-17-S1.doc]

| Description of the Telephone Questionnaire |
| --- |
| Themes Information to verify respondent’s eligibility   - Age of mother - Primiparous - Singleton pregnancy - Term birth - City/town of mother   Characteristics of the mother   - Use and type of oral contraceptive - Breast surgery   Characteristics of the child   - Date of birth - Place of birth   Breastfeeding experience   - Duration - Duration of exclusive breastfeeding - Reasons for breastfeeding cessation   *What were the main reasons you stopped breastfeeding ? *Probe* *3 answers*  *1=*Low milk supply*  *2=*Inconvenienced/tired by breastfeeding*  *3=*Lack of time*  *4=*Nipple pain*  *5=*Breast pain*  *6=*Latching problems/ breast refusal/ baby won’t suck*  *7=*Health problems affecting the mother*  *8=*Infant health status/ weight loss*  *9=*Doctor/health professional’s opinon*  *10=*Partner’s opinon*  *11=*Family/friends’ opinion*  *12=*Return to work/school*  *13=*Infant formula is just as good for baby’s health*  *14=*Desire to drink alcohol*  *15=*Attained breastfeeding objectives*  *16=*Other reason*  *96=*No other*  *77=*DK*  *88=*Refusal*   - Moment when decision to breastfeed was made - Intentions respecting breastfeeding duration - Motivation to overcome difficulties encountered - Main difficulties experienced   *Which of the following problems did you experience?? *Accept* *3 answers*  *1=Nipple pain or injury (chaffing, cracking, vasospasm, thrush on nipple)*  *2=Breast pain or infection (mastitis, thrush on breast, blocked ducts)*  *3=Low milk supply*  *4=Latching problems or breast refusal*  *5=Colic*  *6=Insufficient weight gain*  *7=Sucking difficulties*  *8=Inverted nipples*  *9=*Other problem*  *96=*No other*  *77=*DK*  *88=*Refusal*   - Age when difficulties occurred - Pain experienced - Satisfaction with experience - Introduction of complementary foods   Breastfeeding support   - People who provided the most support   *Which of the following persons gave you the most support and encouragement for continuing to breastfeed? Probe* *3 answers*  *1=Your spouse*  *2=Your mother*  *3=Another family member other than your mother or spouse (sister, sister-in-law, brother, etc.)*  *4=A friend*  *5=Breastfeeding support group*  *6=A midwife*  *7=A physician other than the breastfeeding clinic physician*  *8=nurse from local community services center*  *9=A hospital nurse*  *10=The staff at the Quebec City Breastfeeding Clinic*  *11=*Another person*  *96=*No one else*  *77=*DK*  *88=*Refusal*   - People who hindered breastfeeding - Satisfaction with   - Delivery center services   - Breastfeeding support provided by     - Nurses during home visits     - Support groups     - Physicians   Use of CHUQ breastfeeding clinic   - Number of visits - Child’s age at time of visit(s) - Location of clinic visit - Reasons for consultation - Exclusive breastfeeding before and after visit(s) - Satisfaction with clinic services and staff   *On a scale of 1 to 5, 1 being highly dissatisfied and 5 being highly satisfied, what is your level of satisfaction with the services and interventions provided by the breastfeeding clinic?*  *1=*1, Highly dissatisfied*  *2=*2*  *3=*3*  *4=*4*  *5=*5, Highly satisfied*  *6=*N/A*  *8=*Refusal*   - Respect felt by user - Influence of clinic   - Objectives attained and/or exceeded   *Did consulting the breastfeeding clinic help you reach your breastfeeding goals?*  *1=*Yes*  *2=*No*  *6=*N/A*  *7=*DK*  *8=*Refusal*   - - Increased satisfaction   Other characteristics of the breastfeeding experience   - Cigarette use   Sociodemographic data   - Mother tongue - Marital status - Citizenship - Level of education - Return to work or school - Family income |
