# Supplementary material for: The breastfeeding experience of women with major difficulties who use the services of a breastfeeding clinic: a descriptive study
Source: Int Breastfeed J. 2008 Aug 5;3:17. doi: 10.1186/1746-4358-3-17 (PMC2533286; doi:10.1186/1746-4358-3-17)
Supplement: Additional file 2 — Interview Grid. Wording of questions of the interview. [file 1746-4358-3-17-S2.doc]

| Interview Grid |
| --- |
| **Theme A: Personal breastfeeding experience**  1- Tell me about your breastfeeding experience.  P* - Difficulties encountered  - Breastfeeding duration  2- What motivated you to continue breastfeeding for X weeks?  3- Tell me about how satisfied you feel with your breastfeeding experience.  4- Are you still breastfeeding now? If not, what led you to stop?  P - Reasons related to the mother  - Reasons related to the child  **Theme B: Factors facilitating breastfeeding**    5- What helped you continue breastfeeding?  P - Breastfeeding support service  - Society in general  - Policies  - Personal motivation  6- Are there other things that might have helped you breastfeed longer? If yes, what?    **Theme C: Obstacles and barriers to breastfeeding**  7- Did you encounter any obstacles to breastfeeding? Would you like to tell me about them?  P - Obstacles/barriers  - Something that interfered  **Theme D: Social influence on breastfeeding**  8- Which persons had the most influence on how long you breastfed? How did they influence you?  P - Those who had a positive influence (family, friends, health professionals)  - Those who had a negative influence (family, friends, health professionals)    **Theme E: Experience at the breastfeeding clinic (for Quebec City women only)**  9- How would you describe your experience at the breastfeeding clinic?  P - Type of services received  - Number of visits  10- How did the clinic influence your breastfeeding experience?  P - Through its actions and interventions  - Through the attitude of its staff  11- Aside from the impact on your breastfeeding experience, what other effects did the breastfeeding clinic have?  P - On your family  - Outside your family  12- What did you like most about the breastfeeding clinic?  13- Are there aspects of the clinic that you liked less? If yes, which ones?  P - Negative aspects  - Did the clinic have a harmful influence on you? (breastfeeding, family)  14- How could the clinic better meet your needs?  15- In your view, which breastfeeding clinic services should be maintained and why?  P - Are they useful to other women?  **Theme F: Opinion about the creation of a breastfeeding clinic (For women in Trois-Rivières only)**  *Quebec City has a breastfeeding clinic. Women experiencing serious breastfeeding difficulties are referred to the clinic, where they meet with a physician and a lactation consultant to try to resolve their problems.*  16- What would you think about the usefulness of a breastfeeding clinic in your region?  **Theme G: Other**  17- What else would you would like to add about your breastfeeding experience and its duration?  P - Physical aspects  - Psychological aspects  - Relational aspects |

* P means probe.
